# *Platycodon grandiflorus* polysaccharides deeply participate in the anti-chronic bronchitis effects of *platycodon grandiflorus* decoction, a representative of “the lung and intestine are related”

**DOI:** 10.3389/fphar.2022.927384

**Published:** 2022-09-07

**Authors:** Yang Liu, Qingqing Chen, Rongrong Ren, Qingqing Zhang, Guiming Yan, Dengke Yin, Mingyan Zhang, Ye Yang

**Affiliations:** ^1^ School of Pharmacy, Anhui University of Chinese Medicine, Hefei, China; ^2^ School of Nursing, Anhui University of Chinese Medicine, Hefei, China; ^3^ Anhui Provincial Key Laboratory of Pharmaceutical Preparation Technology and Application, Hefei, China; ^4^ State Key Laboratory of Natural Medicines, China Pharmaceutical University, Nanjing, China

**Keywords:** *Platycodon grandiflorus* polysaccharide, chronic bronchitis, common mucosal immunity, mesenteric lymphatic vessel ligation, Th1/Th2 balance

## Abstract

*Platycodon grandiflorus* (Jacq.) A. DC. (PG) root is one of the most commonly used medicine-food materials for respiratory discomfort in Asia, usually in the form of a decoction or leaching solution. As everyone knows, both of decoction and leaching solution is a polyphase dispersion system, containing low-molecular-weight water-soluble active ingredients and hydrophilic macromolecules. This study aimed to discuss the synergistic effect of *Platycodon grandiflorus* polysaccharide (PGP) and platycodin D (PD) in PG decoction against chronic bronchitis (CB) and the mechanism underlying. A series of PGP, PD, and PGD + PD suspensions were administrated to CB model rats, on the levels of whole animal and *in situ* intestinal segment with or without mesenteric lymphatic vessels ligation. It exhibited that PGP exhibited synergistic effects with PD, on improving the histopathological abnormity, mucus secretion excess, and immunological imbalance in lung of CB model rat, closely associated with its modulations on the mucosal immunity status in small intestine. The polysaccharide macromolecules in PG decoction or leaching solution should be responsible for the modulation of pulmonary immune state, possibly through the common mucosal immune between small intestine and lung. These results might be a new perspective that illustrates the classical theory of “the lung and intestine are related” in traditional Chinese medicine.

## Introduction

Due to dust, air pollution, bacterial or viral infections, allergies, and other risk factors, chronic bronchitis (CB) is becoming a commonly observed respiratory disease, characterized by recurrent attacks and a long course (Kim and Criner, 2013). *Platycodon grandiflorus* (Jacq.) A. DC. (PG) is the sole species of the genus *Platycodon* (Campanulaceae), which is widely grown in Northeast Asia (Zhang et al., 2015) and has been used for hundreds of years as a traditional prescription to relieve cough and eliminate phlegm, among other ailments (Committee for the Pharmacopoeia of PR China, 2020). PG and its compound prescriptions with PG as the principal drug are often used to relieve the symptoms of mucus secretion, cough, expectoration, and gasp (Ryu et al., 2014; Li et al., 2019; Buchwald et al., 2020), in dosage forms of decoction and leaching solution. Studies have demonstrated that the relaxation of platycodin D (PD) in bronchial smooth muscle and its modulatory effects on the immune status of the respiratory tract mucosa are credited with the therapeutic effects of PG (Choi et al., 2001; Kim et al., 2014; Tao et al., 2015; Gao et al., 2017; Hu et al., 2017; Jeon et al., 2019). However, the oral administration process is rapid and impermanent, and the membrane permeability and transmembrane transport efficiency of PD are poor. The results in the literature do not fully elucidate the persistent and targeting effects of PG on the lung.

The decoction and leaching of traditional Chinese medicine (TCM) materials not only dissolve low-molecular-weight water-soluble active ingredients but also harvest abundant biomacromolecules (Ji et al., 2017; Cao et al., 2020; Wu et al., 2020). These are polyphase dispersing systems. After oral administration, the macromolecular or granular immune-active substances in the gut can be endocytosed by microfold cells (M cells) lining the surface of Peyer’s patches (PPs), passed on to lymphoid tissue, absorbed by macrophages, and presented to T and B lymphocytes (Shakweh et al., 2004; Jiang et al., 2019; Kobayashi et al., 2019; Komban et al., 2019). PPs are among the main components of gut-associated lymphoid tissue (GALT). The activated lymphocytes in GALT can migrate to other mucous lymphoid tissues throughout the body, including the bronchial mucosa, through small lymphatic vessels and blood circulation. This immune-sharing mechanism among the mucosa throughout the body is known as the “Common mucosal immune (CMI) mechanism” (Iijima et al., 2001; Uhlig et al., 2004; Kang and Kudsk, 2007; Brandtzaeg, 2010; Tulic et al., 2016). *Platycodon grandiflorus* polysaccharide (PGP) has been shown to have multiple immunomodulatory effects, including the promotion of lymphocyte proliferation, activation, and differentiation and cytokine secretion (Han et al., 2001; Yoon et al., 2003; Yoon et al., 2004; Zhao et al., 2017). Thus, does the mechanism of PG on CB correlate with the activation of PGP on the immune status of the intestinal mucosa and the CMI mechanism between the intestine and lungs?

In TCM, “the lung and intestine are related” is one of the most important theories. Based on this theory, there are several unique therapeutic methods and principles for lung diseases in TCM clinics, namely “Treating the intestine and the lung simultaneously,” “Treating the intestine for lung diseases,” and “Treating lung diseases through the intestine.” PG is one of the most commonly used TCM materials for the treatment of lung disease. This study aimed to discuss the therapeutic mechanism of PG decoction or leaching solution on BC based on the polyphase dispersion system of PD and PGP and the CMI between the intestine and lung using a combination of an *in vivo* intestinal perfusion model and mesenteric lymphatic vessel ligation. The results of this study should be a new perspective that illustrates the classical theory of TCM.

## Materials and methods

### Chemical reagent

PGP (purity ≥98%) and PD (purity ≥98%) were purchased from Desite Biotechnology Co. Ltd. (Chengdu, China). The ambroxol hydrochloride oral solution was purchased from Sunflower Pharmaceutical Group Co., Ltd. (Hengshui, China). Rabbit anti-rat antibodies specific to interferon-γ (IFN-γ, bs-0480R), interleukin-4 (IL-4, bs-0581R), mucin 2 (MUC2, bsm-60016R), endothelin-1 (ET-1, bs-0954R), inducible nitric oxide synthase (iNOS, bs-2072R), T-bet (bs-3599R), GATA-3 (bs-1452R), polymer detection system kit (PV-6000), 3, 3′-diaminobenzidine (DAB), and horseradish peroxidase (HRP) were purchased from Beijing Biosynthesis Biotechnology Co., Ltd. (Beijing, China). Enzyme-linked immunosorbent assay (ELISA) kits for IFN-γ and IL-4 were purchased from Abcam (Cambridge, UK). The Alcian blue/periodic acid-Schiff (AB-PAS) stain kit and nitric oxide (NO) analysis kit were purchased from Beijing Solarbio Science &Technology Co., Ltd. (Beijing, China). Antibodies of FITC anti-rat CD4 (201,505), Alexa Fluor® 647 anti-rat IFN-γ (507,809), and PE anti-rat IL-4 (511,905) were purchased from BioLegend (California, United States). All other chemicals and solvents were of analytical grade or higher and were purchased from Sinopharm Chemical Reagent Co., Ltd. (Shanghai, China).

### Animals

Sprague-Dawley rats (SD rats; male; 180–200 g; 5 months old) were obtained from the Experimental Animal Center of Anhui Medical University (certificate number SCXK 2017–001, Anhui, China). All animals were housed in a specific pathogen-free environment with a 12 h light-dark cycle and provided free access to a standard laboratory diet and water. All animal operational experiments were strictly in line with the P. R. China legislation on the use and care of laboratory animals and were approved by the Animal Care Review Committee of the Anhui University of Chinese Medicine.

### Induction of chronic bronchitis in rat and drug administration

**FIGURE 1:**
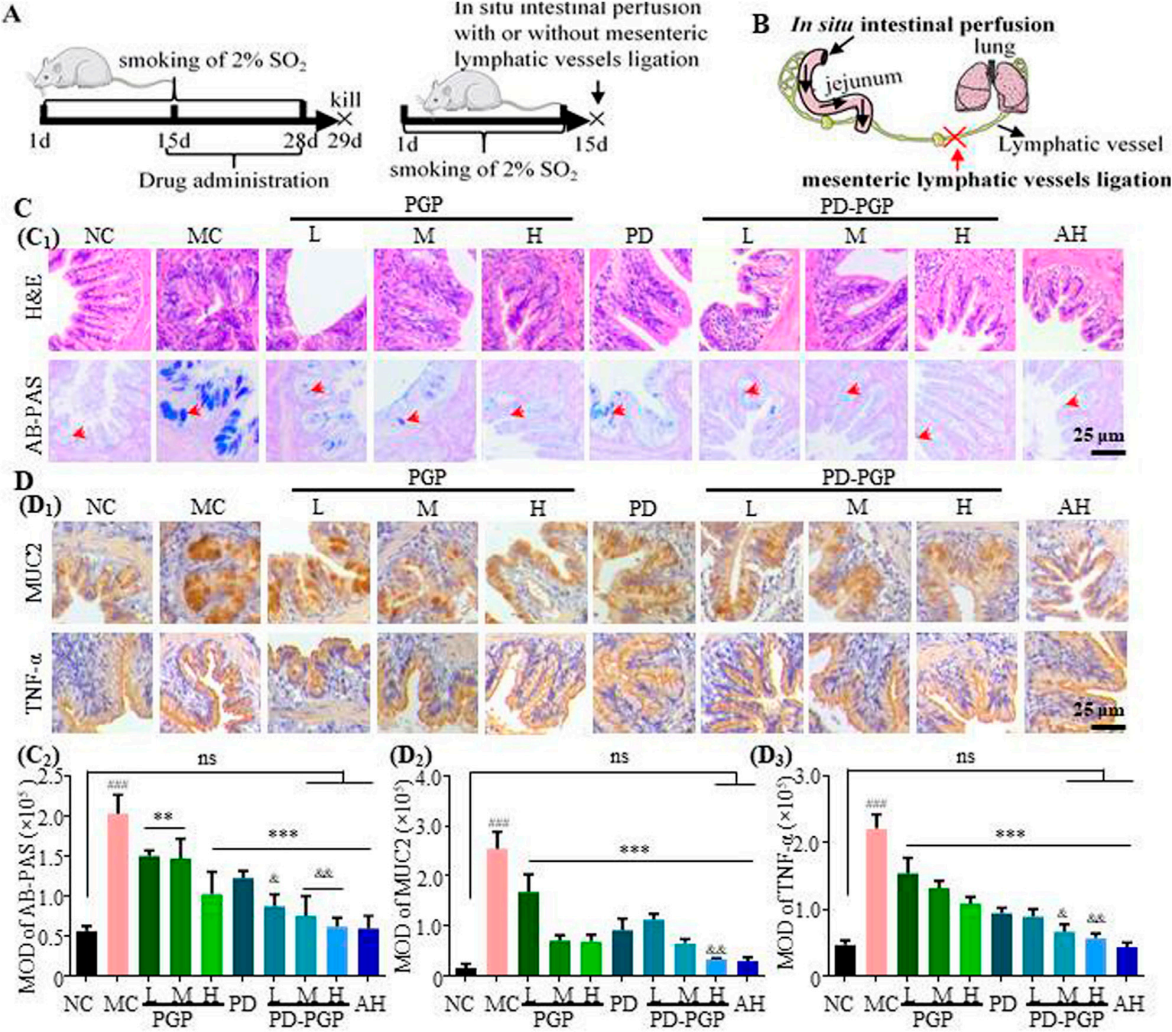
Synergistic effects of PD and PGP on ameliorating structural abnormality, excessive mucin secretion, and inflammatory state of the lung in CB rats. **(A)** Experimental design of 2% SO_2_-induced CB model and drug treatment. **(B)** Illustration of a combination of *in situ* intestinal perfusion and mesenteric lymphatic ligation. **(C)** Representative H&E-staining and AB-PAS staining images (C1) and MOD analyses (C2) of lung tissues after administration with low/medium/high dose of PGP (75, 150 and 300 mg/kg, groups PGP_L/M/H_), PD (2 mg/kg, group PD), PD + low/medium/high dose of PGP (groups PD-PGP_L/M/H_), and positive drug ambroxol hydrochloride (8.1 mg/kg, group AH), with the lung from groups NC and MC as a comparison. **(D)** Representative images (D1) and MOD analyses (D2 and D3) of IHC staining MUC2 and TNF-α expression in lung tissues after administration with PGP, PD, and PD-PGP, with the lung from groups NC and MC as a comparison [Original magnification ×200. Red arrow, acidic mucus. Data are expressed as mean ± SD, with data obtained from 10 randomly selected fields. Data are expressed as mean ± SD (*n* = 6); ^**^ *p* < 0.01 and ^***^ *p* < 0.001 versus MC group; ^###^ *p* < 0.001 versus NC group; ^&^ *p* < 0.05, ^&&^ *p* < 0.01 and ^&&&^ *p* < 0.001 versus PD group].

The CB rat model was induced by daily smoking with 2% (v/v) sulfur dioxide (SO_2_) for 30 min according to a previous report (Nikula and Green, 2000). After 15 days of smoking, the model rats were randomly grouped and gavaged with solutions of normal saline (group MC), low/medium/high dose of PGP (75, 150, and 300 mg/kg; groups PGP_L/M/H_), PD (2 mg/kg, group PD), PD + low/medium/high dose of PGP (groups PD-PGP_L/M/H_), and the positive drug ambroxol hydrochloride (8.1 mg/kg, group AH) before SO_2_ exposure. The dosage of PD and PD + PGP were determined according to the dosage of PG specified in Pharmacopoeia of PR China and the content of PGP and PD in PG (Committee for the Pharmacopoeia of PR China, 2020). Rats gavaged with normal saline were used as the NC group. After continuing smoking and drug administration for 15 days, the rats were sacrificed, and lung tissues were collected for subsequent experiments. A flow diagram of these processes is shown in Figure 1A.

### Histopathological examination of lung tissues

Lung tissues in each group were fixed and sliced into 5 μm sections. Hematoxylin and eosin (H&E) staining was performed to evaluate histomorphological features. AB-PAS staining was performed to evaluate mucin secretion, distribution, and type. Immunohistochemistry (IHC) staining was performed to evaluate the secretion of MUC2, TNF-α, iNOS, and ET-1. Ten areas under ×200 field of vision were randomly selected, and the mean optical density (MOD) was calculated using Image-pro Plus 6.0, through deviding the intergral optical density of nucleus into the intergral optical density of positive expression. The NO level in lung tissue homogenates was detected using a Micro NO Content Assay Kit (Beijing Solarbio Science &Technology Co., Ltd., Beijing, China), according to previous report (Gong et al., 2022). In brief, tissue samples were precisely weighed about 0.2 g, added into 1.0 ml of extract solution, homogenized in an ice bath, and centrifuged at 12,000 rpm for 15 min at 4°C. The supernatants were determine the absorbance at 550 nm, and the NO content was calculated according to the standard curve of standards. The ET-1 level in lung tissue homogenates was detected using a ET-1 ELISA kit (JL10931, Shanghai Jianglai Biotechnology Co., LTD., Shanghai, China), according to the product instruction. In brief, the standards or sample homogenates were added into the anti-ET-1 antibody coated plate, washed away the free materials, bound with the biotinylated secondary antibody and avidin-HRP, coloured with 3,3′,5,5′-tetramethyl-benzidine, and then determined the absorbance at 450 nm.

### Correlation study on common mucosal immune between small intestine and lung during *Platycodon grandiflorus* polysaccharide administration, through a combination of *in situ* intestinal perfusion and mesenteric lymphatic ligation

*In situ* circulated intestinal perfusion was performed on CB rats after 15 days of SO_2_ smoking to evaluate the influence of PGP on the mucosal immunity status of the small intestine and lung, according to literatures (Lozoya-Agullo et al., 2015). In brief, after intraperitoneal anesthetization by pentobarbital sodium (50 mg/kg), the CB rats were first fixed on the operation table, enterocoelia were opened along the middle line of the abdomen, and the small intestine was exposed. Next, approximately 10 cm of the jejunum was inserted with a rubber tube in 3 mm diameter, ligated on both sides, and the intestinal contents were rinsed by perfusion with normal saline. A series of Kreb-Ringer’s (K-R) solutions (100 ml) containing low/medium/high doses of PGP (1, 2, and 4 mg/ml, groups PGP_L/M/H_), PD (20 μg/ml, group PD), and PD + high dose of PGP (groups PD-PGP_H_) were circularly perfused into the jejunal segment with a flow rate of 1.0 ml/min, using a peristaltic pump (LONGER YZ1515x, Longer Precision Pump Co., Ltd., Hebei, China). Normal and CB rats circularly perfused with K-R solution were set as the NC and MC groups, respectively. In addition, one group of CB rats was subjected to a combination of *in situ* intestinal perfusion and mesenteric lymphatic vessel ligation (as shown in Figure 1B), according to literatures (Watkins et al., 2008; Tong et al., 2016), and named as group PGP_H_/Lig. In brief, after double-sided intubation and rinsing of the intestinal contents, the mesenteric lymphatic vessels of this jejunal segment were separated and ligated, and the K-R solution with a high dose of PGP was circularly perfused as described aforementioned. Another group of CB model rats underwent the same operations as aforementioned, were threaded with a ligature under the mesenteric lymphatic but without ligation, then circularly perfused with K-R solution, and named as group Sham. After 90 min of perfusion, the rats in each group were sacrificed and immersed in 75% ethanol for 5 min, and the PPs from jejunum and lung tissues were collected for subsequent detection.

The differentiation of T lymphocytes into Th1 and Th2 cells in PPs and lungs was detected using flow cytometry. For flow cytometry, PPs and lung tissues were gently crushed with the base of a sterile syringe, re-suspended, filtered with a 200-mesh cell screen, and lysed red blood cells. After counting, 1.0 × 10^6^ cells were collected and stained with fluorescently labeled FITC-CD4, Fluor® 647-IFN-γ, and PE-IL-4 and analyzed using a Beckman Coulter flow cytometer (Beckman Coulter, FC500-MPL). The expression levels of IFN-γ and IL-4 in PPs and lung tissues were measured using ELISA. All procedures were performed according to the manufacturer’s instructions.

The correlation of transcription factor levels, T lymphocyte differentiation, and immune effector secretion between PPs and lung tissues was performed by Pearson correlation analysis using SPSS 25.0. The relevant values of T lymphocyte differentiation, transcription factor expression, and cytokine secretion in PPs and lungs were imported into the software, and the Pearson correlation coefficient in double correlation was used for two-sided tests.

### Statistical analysis

All data are presented as the mean ± SD (*n* = 6). Statistical analysis was conducted by one-way analysis of variance followed by the LSD post hoc test, with *p* < 0.05, 0.01, or 0.001 considered significant or extremely significant differences.

## Results

### Synergistic effects of platycodin D and *Platycodon grandiflorus* polysaccharide on ameliorating histological abnormality in chronic bronchitis rats

Figure 1C shows no obvious hyperplasia, thickening, or inflammatory exudation of the lung tissue in the NC group. There was no edema fluid or hemorrhage in the bronchial cavity or alveoli at any level. The lung tissue structure in the CB model (MC group) showed obvious proliferation and invasion. Inflammatory cell infiltration and fibrous tissue proliferation were observed in bronchiolar mucosa. After drug administration, PD and PD-PGP improved lung tissue damage and reduced the inflammatory exudation. The combination of PGP and PD showed stronger effect than that of PD.

Increased mucus secretion, mucin expression, and inflammatory response in the lungs are indicators of the severity of CB (Andelid et al., 2021). Figure 1C shows that the acidic mucus (red arrow) in the lung tissues of the MC group increased significantly. After gavage with PD/PD-PGP, the secretion of acidic mucous in the lung tissues was reduced significantly, similar to that in the NC group. Figures 1D1,D2 shows that the expression of MUC2 in the lung tissues in the MC group was significantly increased. After gavage with PD/PD-PGP, the expression of MUC2 in lung tissue was significantly reduced, similar to that in the NC group. The inhibitory effects of each PD-PGP group on MUC2 secretion were more significant than those in the PD group (*p* < 0.001). The results in Figures 1D1,D3 show that the inflammatory factor TNF-α was significantly increased in the lung tissue of the MC group. After gavage with PD/PD-PGP, TNF-α expression in CB rats was significantly inhibited, and the inhibitory effect in each PD-PGP group was more obvious than that in the PD group (*p* < 0.001).

### Synergistic effects of platycodin D and *Platycodon grandiflorus* polysaccharide on regulating abnormal pulmonary vasoconstriction and vasodilation in chronic bronchitis rats

**FIGURE 2:**
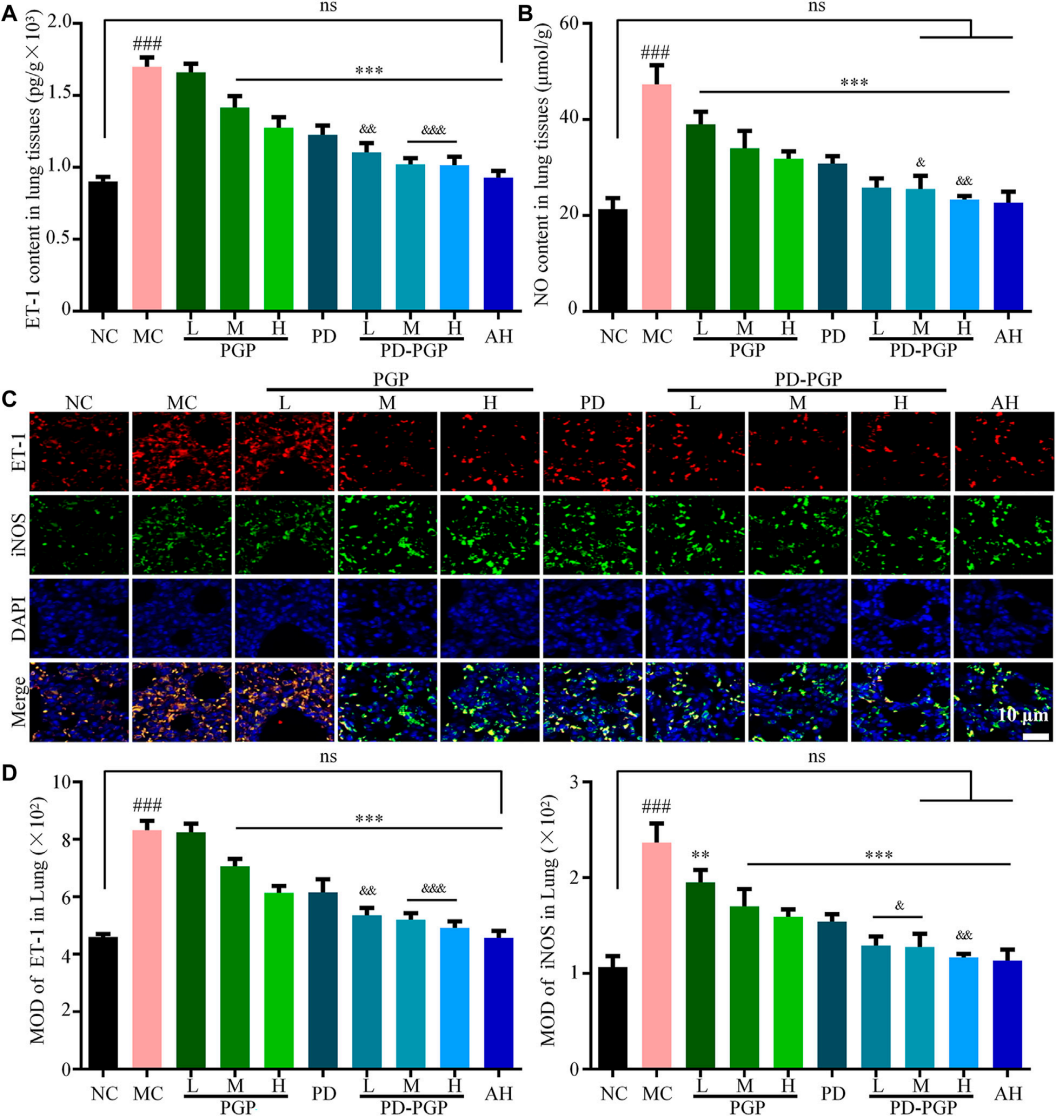
Synergistic effects of PD and PGP on alleviating the abnormal vasoconstriction and vasodilation of lung in CB rats. **(A,B)** Detection of ET-1 **(A)** and NO **(B)** contents in lung tissues after administrated with PGP (groups PGP_L/M/H_), PD (group PD) and PD-PGP (groups PD-PGP_L/M/H_), with the lung tissues from groups NC and MC as comparison and the lung tissues from group AH as positive control. **(C,D)** Representative images **(C)** and MOD analyses **(D)** of IHC staining ET-1 and iNOS expression in lung tissues after administrated with PGP, PD and PD-PGP, with the lung tissues from groups NC and MC as comparison and the lung tissues from group AH as positive control. (Original magnification ×200. Data are expressed as mean ± SD, with data obtained from 10 randomly selected fields. Data are expressed as mean ± SD (*n* = 6); ^**^ *p <* 0.01 and ^***^ *p <* 0.001 *versus* MC group; ^###^ *p <* 0.001 *versus* NC group; ^&^ *p <* 0.05, ^&&^ *p <* 0.01 and ^&&&^ *p <* 0.001 *versus* PD group).

ET-1 and NO are involved in the vasoconstriction and vasodilation of lungs, respectively. Figures 2A,B shows that, the ET-1 and NO contents and the ET-1 and iNOS expression in the lung tissue of the MC group were significantly higher than those of the NC group (*p <* 0.001). After gavage with PD/PD-PGP, the ET-1 and NO levels and ET-1 and iNOS expression in lung tissue were significantly reduced, similar to those in the NC group, as shown in Figures 2C,D. The inhibitory effect in each PD-PGP group was more significant than that in the PD group (*p <* 0.001). These results indicated a synergistic effect of PD and PGP on abnormal pulmonary vasoconstriction and vasodilation in CB rats.

### *Platycodon grandiflorus* polysaccharide adjusts immune imbalance in lung tissues of chronic bronchitis rats through common mucosal immune between intestine and lung.

**FIGURE 3:**
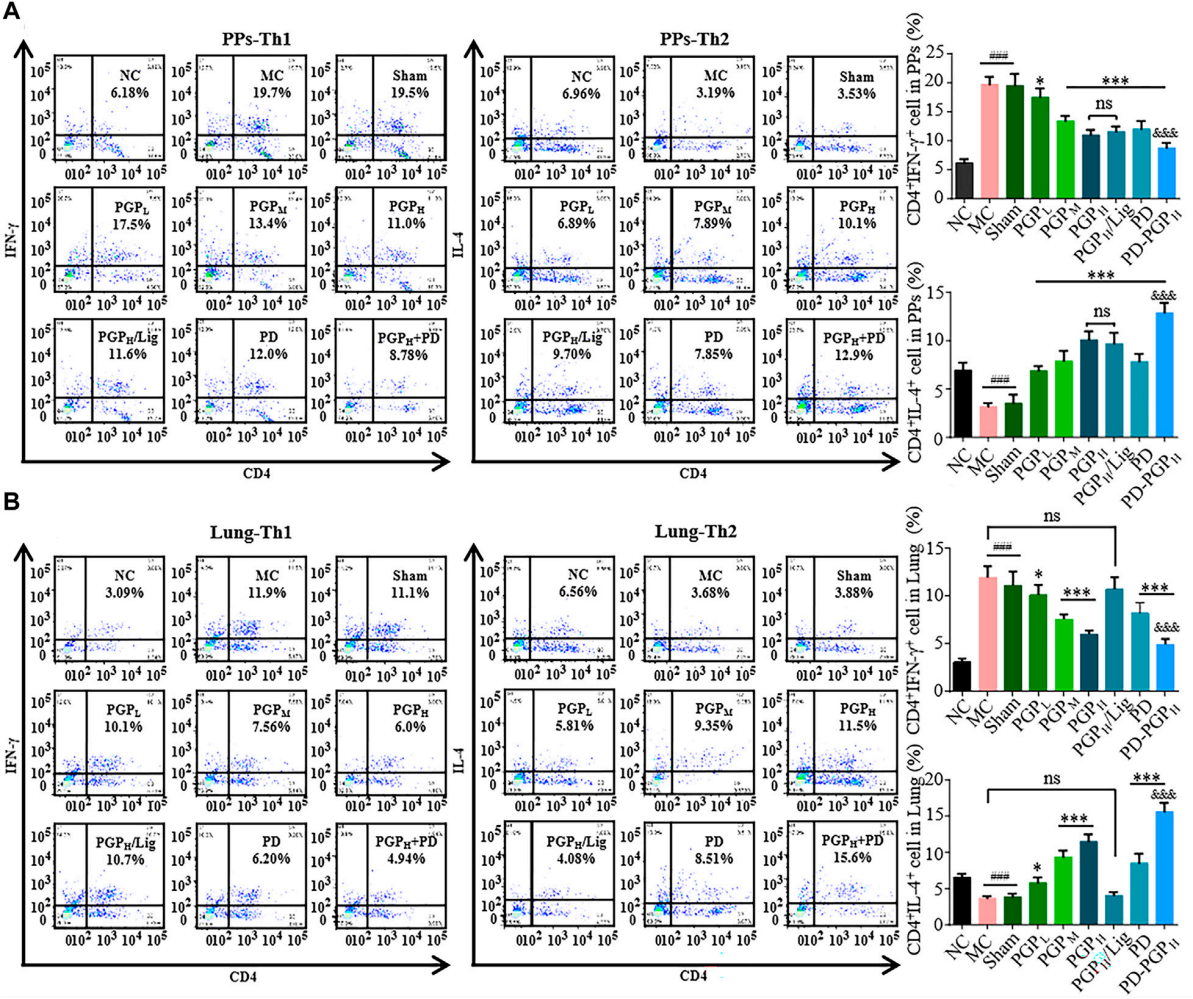
Flow cytometry scatter plots (A1 and B1) and data analysis charts (A2 and B2) of Th1 (CD4^+^IFN-γ^+^) and Th2 (CD4^+^IL-4^+^) proportions in PPs **(A)** and lung tissues **(B)** of CB rats, after 90 min intestinal perfusion of PGP (groups PGP_L/M/H_), PD (group PD), and PD-PGP (groups PD-PGP_H_), with (group PGP_H_/Lig) or without mesenteric lymphatic vessel ligation. Th1 and Th2 proportions in PPs and lung tissues from normal (groups NC), model (group MC), and sham-operated (group Sham) rats were set as the comparison. (Data are expressed as mean ± SD (*n* = 6); ^*^ *p <* 0.05 and ^***^ *p <* 0.001 *versus* MC group; ^###^ *p <* 0.001 *versus* NC group; ^&&&^ *p <* 0.001 *versus* PD group).

As shown in Figure 3, the *in situ* perfusion of PGP, PD, and PGP-PD can significantly adjust the Th1/Th2 imbalance, reducing the Th1 proportion and increasing the Th2 proportion in the PPs and lung tissues of BC model rats, and the sham threading of ligature under mesenteric lymphatic vessels exhibited no effect. However, after mesenteric lymphatic vessel ligation, the regulatory effect of PGP on the Th1/Th2 balance in lung tissues disappears. These results suggest that the CMI between the small intestine and lung is a physiological basis for the regulation of Th1/Th2 imbalance by PGP.

**FIGURE 4:**
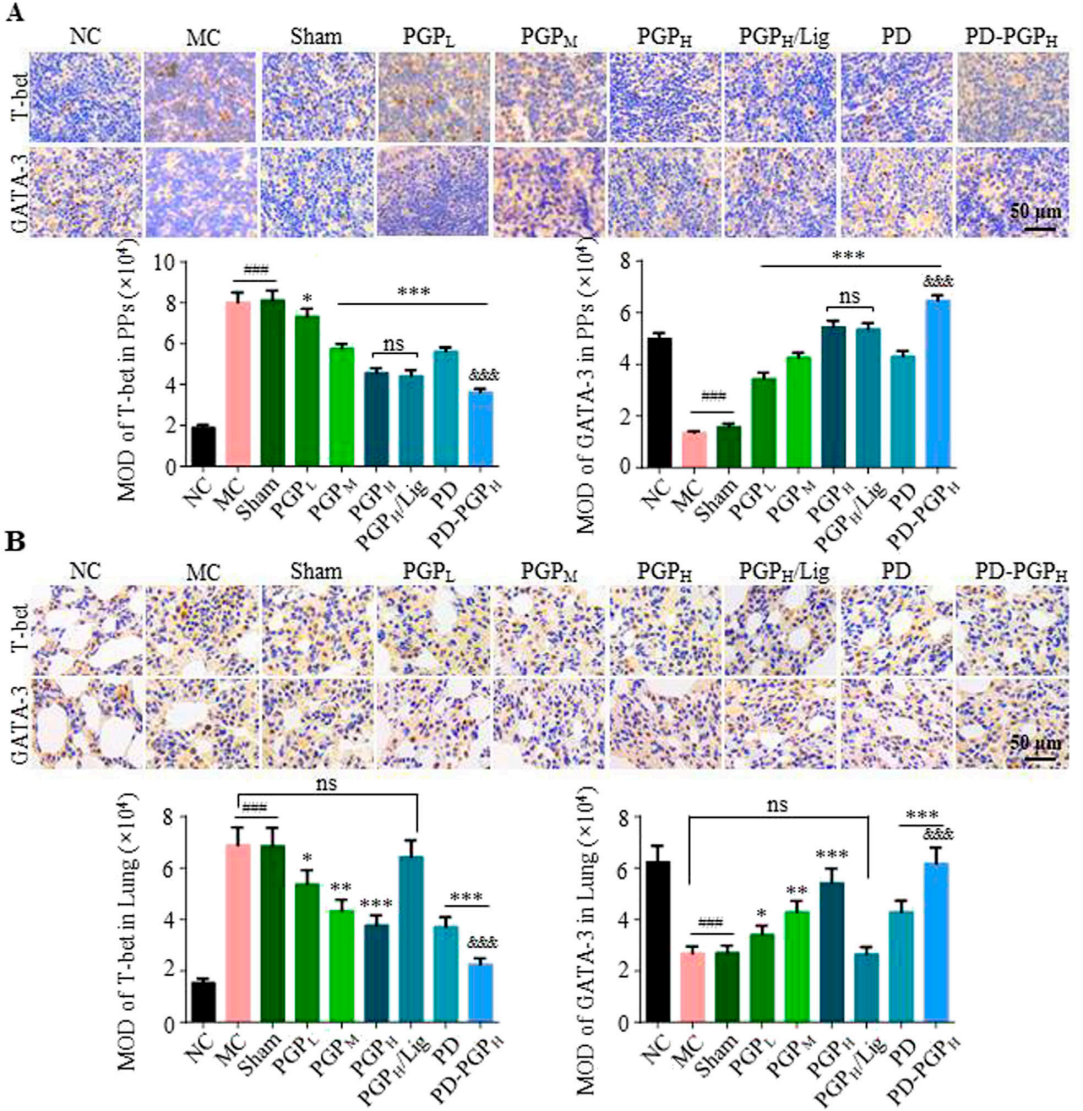
Representative images (A1 and B1) and MOD analyses (A2 and B2) of IHC staining T-bet and GATA-3 expression in PPs **(A)** and lung tissues **(B)** of CB rats, after 90 min intestinal perfusion of PGP (groups PGP_L/M/H_), PD (group PD), and PD-PGP (groups PD-PGP_H_), with (group PGP_H_/Lig) or without mesenteric lymphatic vessel ligation. T-bet and GATA-3 expression in PPs and lung tissues from normal (groups NC), model (group MC), and sham operated (group Sham) rats were set as the comparison (Original magnification ×200. Data are expressed as mean ± SD, with data obtained from 10 randomly selected fields. Data are expressed as mean ± SD (*n* = 6); ^*^ *p* < 0.05, ^**^ *p* < 0.01 and ^***^ *p* < 0.001 versus MC group; ^###^ *p* < 0.001 versus NC group; ^&&&^ *p* < 0.001 versus PD group).

T-bet and GATA-3 are nuclear transcription factors specific for Th1 and Th2 cells. As shown in Figure 4, PGP and PD, and the combination of the two, significantly improved the abnormal T-bet increase and GATA-3 decrease in PPs and lung tissues of CB model rats. After mesenteric lymphatic vessel ligation, these regulatory effects of PGP still exist in PPs but disappear in the lungs.

**FIGURE 5:**
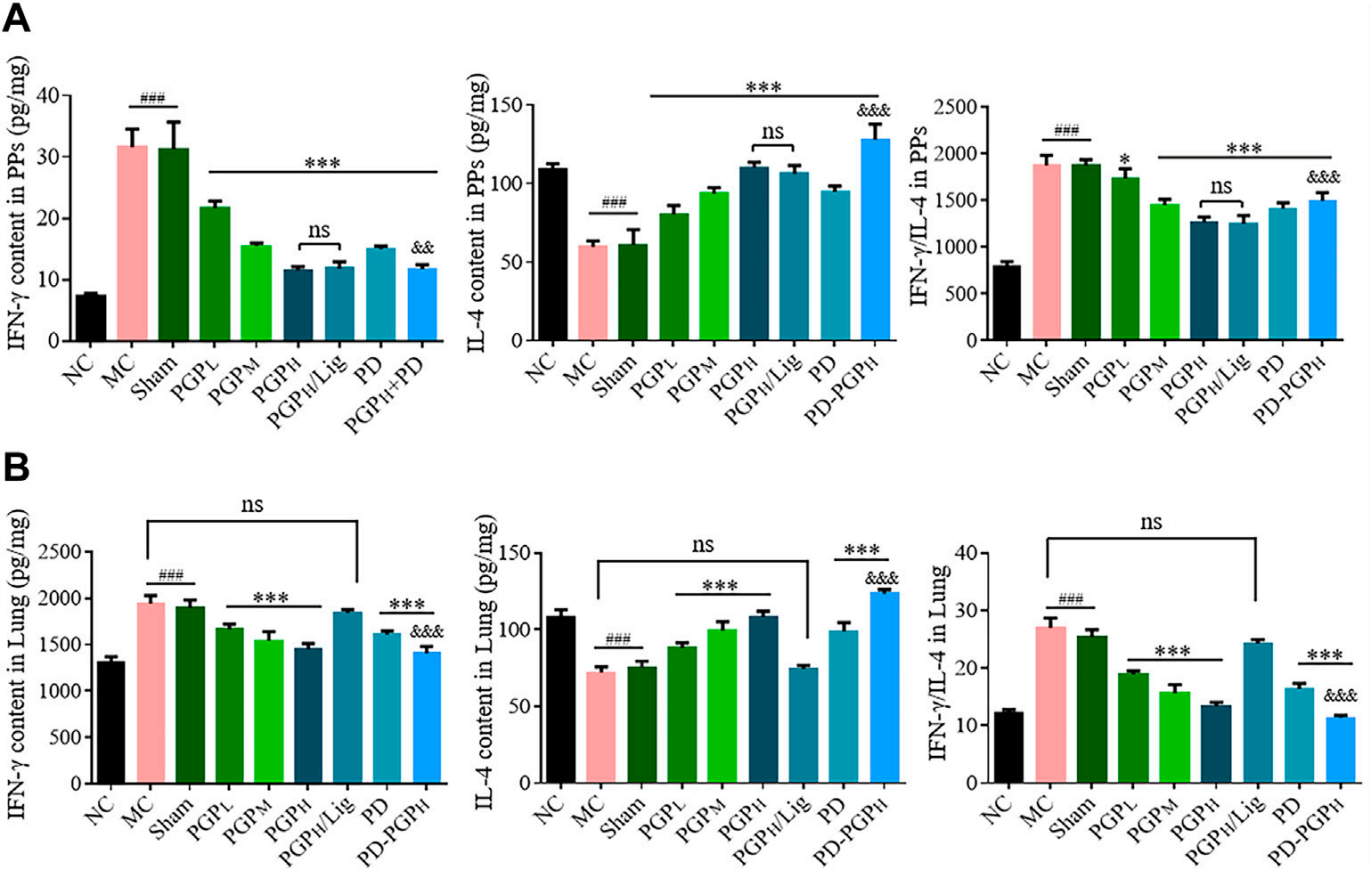
ELISA results (A1 and B1) and ratios analysis (A2 and B2) of IFN-γ and IL-4 contents in the PPs **(A)** and lung tissues **(B)** of CB rats, after 90 min intestinal perfusion of PGP and PD (groups _*situ*_PGP_L/M/H_, _*situ*_PD, _*situ*_PD-PGP_H_), with (group _*situ*_PGP_H_/Lig) or without mesenteric lymphatic vessel ligation. IFN-γ and IL-4 contents in PPs and lung tissues from normal (groups _*situ*_NC), model (group _*situ*_MC), and sham-operated model (group Sham) rats were set as the comparison. (Data are expressed as mean ± SD (*n* = 6); ^*^ *p <* 0.05 and ^***^ *p <* 0.001 *versus* MC group; ^###^ *p <* 0.001 *versus* NC group; ^&&&^ *p <* 0.001 *versus* PD group).

Figure 5 shows that the administration of PGP, PD, and PD-PGP can improve the abnormal increase in IFN-γ secretion and decrease IL-4 secretion in PPs and lung tissues of CB model rats, dose-dependently correlated with the PGP concentration, and significantly adjust the IFN-γ/IL-4 imbalance in lung tissue. However, these effects were not observed in the lung tissues after mesenteric lymphatic vessel ligation.

### Correlation analysis of intestinal and pulmonary immune state during *Platycodon grandiflorus* against chronic bronchitis

**FIGURE 6:**
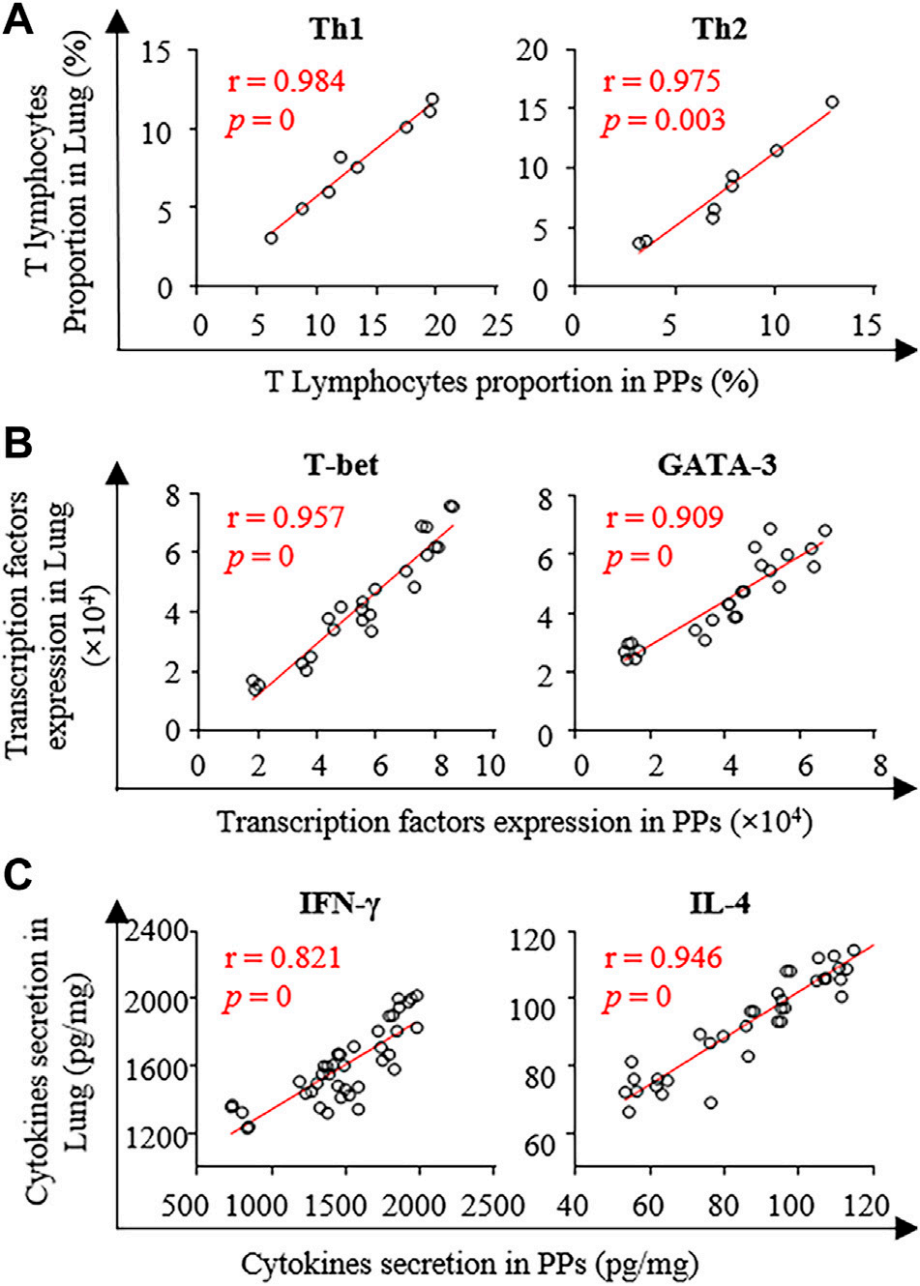
Pearson analysis of the T lymphocytes differentiation **(A)**, transcription factors expression **(B)**, and cytokines secretion **(C)** between PPs and lung.

As shown in Figure 6, all correlation coefficients *r* of T lymphocyte differentiation, transcription factor expression, and cytokine secretion in PPs and lungs were greater than 0.8. These results indicate a strong positive correlation between the pulmonary and intestinal immune states during PG administration in the CB.

## Discussion

The theory of “The lung and the intestine are related” is crucial in the clinical practice of TCM, however, it springs from the earliest classical works of *Huangdi Emperor’s Internal Classic* in China, which was thought to be created in Pre-Qin to Han dynasties (from 221 BC to 220 AD). In the Zang-fu theory in TCM, the “five Zang-organs” and the “six Fu-organs” are considered to be the description of functions but not to be equal to the anatomical structure in modern medicine. In TCM, the function of small intestine is bearing, digestion, secretion, and distinguish the Qingzhuo; large intestine is an organ in charge of transportation, that is eliminate the food residue from the body and re-absorb the body fluid. Thus, the large intestine in TCM should include the structure and function of colon, rectum, and part of small intestine in modern medicine. And this theory is also been described as “The lung and the large intestine are related”. It is implemented not only in the analysis of pathogenesis and syndromes of lung and intestine diseases but also in the application of treatment principles (Liu et al., 2012; Ni and Gao, 2012). Many modern medical studies have confirmed this theory from the correlation analysis of structural and functional changes between lung and intestinal tissues in the occurrence and outcome of related diseases (Ding et al., 2020; Lee et al., 2021). However, few systematic and scientific studies have focused on explaining the pharmaceutical effects of Chinese medicine based on this theory.

CB is a common respiratory disease and TCM has curative effects. PG is commonly used in CB. The use of PG alone and compound prescriptions for respiratory diseases has a history of thousands of years in China (Buchwald et al., 2020; Deng et al., 2020; Deng et al., 2021). Studies have shown that PG inhibits the secretion of mucus and regulates the differentiation of immune cells and the expression of inflammatory factors (Kim et al., 2011; Huang et al., 2012; Lee et al., 2019; Sun et al., 2020). Modern medical research has confirmed that the saponin of PD in PG can inhibit airway mucin secretion in the inflammatory state (Shin et al., 2002; Ryu et al., 2014). However, there has been little development and application of PD preparation, and PG remains mostly used in the form of “decoction” or “aqueous leaching” (Choi et al., 2009; Lee et al., 2020). These phenomena suggest that the role of PG in respiratory diseases should not be completely attributed to PD alone but to the synergistic effect of multiple components in PG. We presume that there should be some other unknown mechanism of the PG decoction or leaching on relieving the respiratory discomfort. That is the aim of this study, to discuss the advantages of the traditional dosage form of decoction and leaching solution in relieving respiratory symptoms.

Previous studies have reported the promotion of PGP on CD4^+^ T cell level (Zhao et al., 2017). PD is considered to be a potential immunologic adjuvant, which can significantly enhance the immunogenicity of vaccine (Xie et al., 2008) and promote the expression of Th2 cytokines (IL-4 and IL-10) and transcription factors (GATA-3) (Xie et al., 2008; Xie et al., 2010; Xie et al., 2010). In the present study, the combination of PD and PGP exhibited a significant synergistic effect on Th2 differentiation, thus the CD4^+^IL4^+^ cells level was even higher than that in NC group. The mucosa-associated lymphoid tissues, cells, and effector factors in different organs form a complex network called the CMI system (Kang and Kudsk, 2007; Tulic et al., 2016). It is also the theoretical basis of the multiple organ dysfunction syndrome in patients with abdominal infection and intestinal ischemia-reperfusion injury (Deitch, 2010; Deitch, 2012). Oral vaccines against rotavirus, poliovirus, *Vibrio cholera*, and *Salmonella typhi* are developed based on CMI system (Azegami et al., 2014; Kiyono and Azegami, 2015). Because of the anatomical structure, lung is the first organ exposure to the intestinal inflammatory mediator (Mjösberg and Rao, 2018). So, the immune correlation between the lung and intestinal mucosa is also considered the material basis of the theory of “The lung and the intestine are related” in TCM (Liu et al., 2012; Tulic et al., 2016). In inflammatory bowel disease and chronic obstructive pulmonary disease, the activated lymphocytes in the intestine or lung can migrate to other mucosal sites through lymphatic circulation (Spahn and Kucharzik, 2004; Keely et al., 2012; Kim and Song, 2017). Many polysaccharides in TCM have been proven to have multiple immunoactivities, such as the activation of dendritic cells, macrophages, and B lymphocytes (Park et al., 2014; Zhao et al., 2017; Wu et al., 2018; Ying et al., 2020). Because of their macromolecular and polyhydroxy characteristics, polysaccharide chains commonly aggregate at different levels in some liquid-phase systems (Cesàro et al., 2012; Kontogiorgos, 2019; Yi et al., 2020). Our prior study showed that polysaccharides can aggregate in solution (Chen et al., 2021). Macromolecular aggregates can be ingested by intestinal-related lymphoid tissues and are present in T and B lymphocytes (Randolph et al., 2017; Johnson, 2021). Our study confirmed the blocked bronchial lymphocyte proportion adjustment after mesenteric lymphatic vessel ligation (Figure 3). These results could be compelling evidence that the immune-modulating function of PGP in CB is mediated through the CMI system between the intestine and lung, and the classic theory of “The lung and intestine are related” in TCM.

Our results suggest that the polysaccharide macromolecule PGP plays a crucial role in the clinical use of PG in CB therapy through the CMI system-mediated modulatory effects on the pulmonary immune state. This finding might provide some experimental and theoretical bases for the theory of “The lung and the intention are related” in TCM and guide its clinical application. Nevertheless, it can hardly be denied that there is much more to be said about this theory and our hypothesis still need further analysis and meticulous argumentation, especially the further tests about mucous secretion, pulmonary vasoconstriction/vasodilation, and homing of lymphocyte.

## Data Availability

The raw data supporting the conclusion of this article will be made available by the authors, without undue reservation.
