# Corrigendum: *Platycodon grandifloras* polysaccharides deeply participate in the anti-chronic bronchitis effects of *platycodon grandiflorus* decoction, a representative of “the lung and intestine are related”

**DOI:** 10.3389/fphar.2025.1566123

**Published:** 2025-04-30

**Authors:** Yang Liu, Qingqing Chen, Rongrong Ren, Qingqing Zhang, Guiming Yan, Dengke Yin, Mingyan Zhang, Ye Yang

**Affiliations:** ^1^ School of Pharmacy, Anhui University of Chinese Medicine, Hefei, China; ^2^ School of Nursing, Anhui University of Chinese Medicine, Hefei, China; ^3^ Anhui Provincial Key Laboratory of Pharmaceutical Preparation Technology and Application, Hefei, China; ^4^ State Key Laboratory of Natural Medicines, China Pharmaceutical University, Nanjing, China

**Keywords:** *platycodon grandiflorus* polysaccharide, chronic bronchitis, common mucosal immunity, mesenteric lymphatic vessel ligation, Th1/Th2 balance

In the published article, there was an error in Figure 1 as published. In Figure 1D, there was a picture misuse of IHC staining MUC2 between group PGP_H_ and group AH. The corrected Figure 1 and its caption appear below.

**FIGURE 1:**
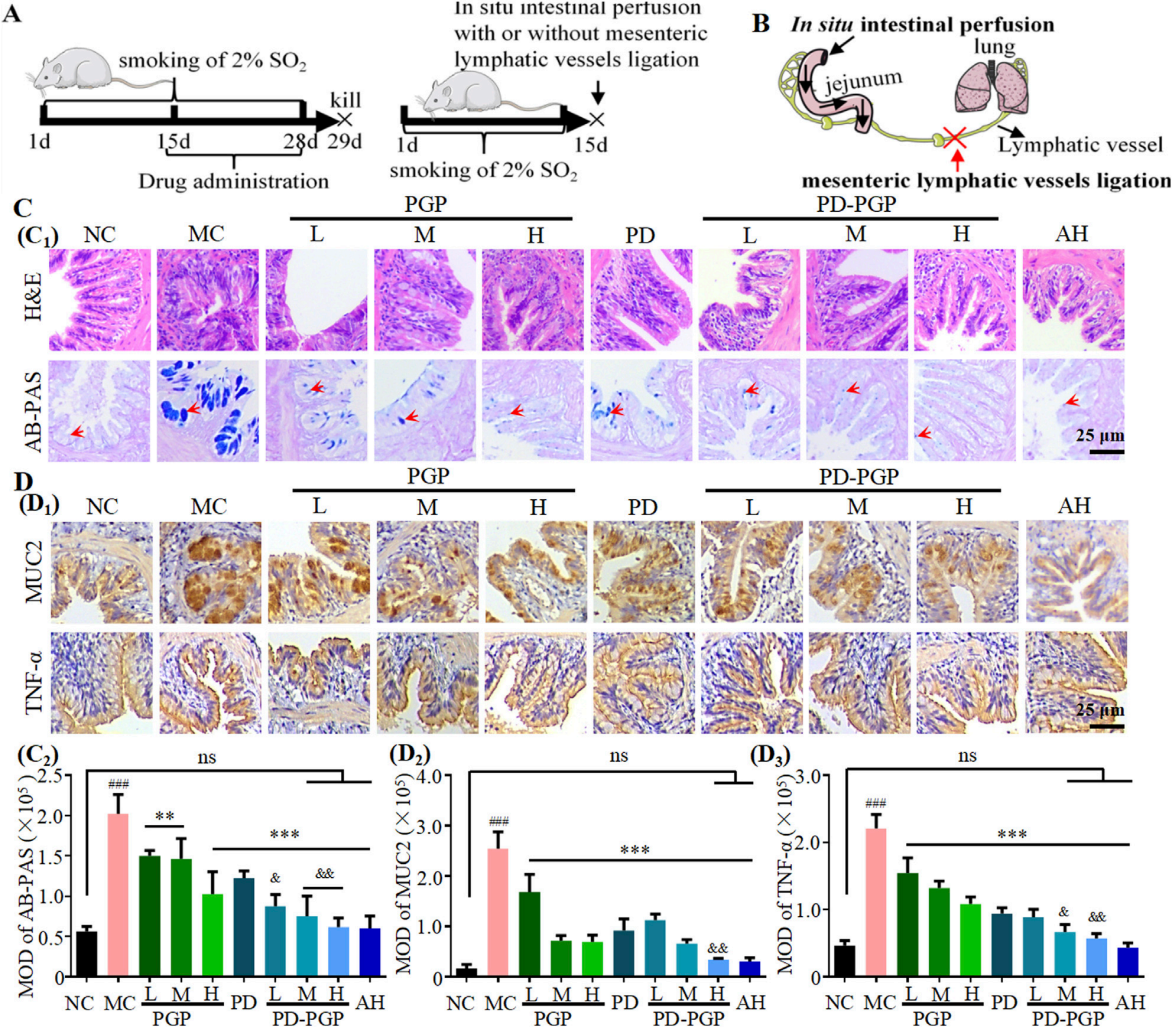
Synergistic effects of PD and PGP on ameliorating structural abnormality, excessive mucin secretion, and inflammatory state of the lung in CB rats. **(A)** Experimental design of 2% SO_2_-induced CB model and drug treatment. **(B)** Illustration of a combination of *in situ* intestinal perfusion and mesenteric lymphatic ligation. **(C)** Representative H&E-staining and AB-PAS staining images (C1) and MOD analyses (C2) of lung tissues after administration with low/medium/high dose of PGP (75, 150 and 300 mg/kg, groups PGP_L/M/H_), PD (2 mg/kg, group PD), PD + low/medium/high dose of PGP (groups PD-PGP_L/M/H_), and positive drug ambroxol hydrochloride (8.1 mg/kg, group AH), with the lung from groups NC and MC as a comparison. **(D)** Representative images (D1) and MOD analyses (D2 and D3) of IHC staining MUC2 and TNF-α expression in lung tissues after administration with PGP, PD, and PD-PGP, with the lung from groups NC and MC as a comparison [Original magnification ×200. Red arrow, acidic mucus. Data are expressed as mean ± SD, with data obtained from 10 randomly selected fields. Data are expressed as mean ± SD (*n* = 6); ^**^ *p* < 0.01 and ^***^ *p* < 0.001 versus MC group; ^###^ *p* < 0.001 versus NC group; ^&^ *p* < 0.05, ^&&^ *p* < 0.01 and ^&&&^ *p* < 0.001 versus PD group].

In the published article, there was an error in Figure 4 as published. In Figure 4A, picture duplication occurred in T-bet IHC staining analyses between group PGPL and group MC, group PD-PGPH and group NC, group PD and group PGPH/Lig, and GATA-3 staining analyses between group MC and group Sham. The corrected Figure 4 and its caption appear below.

**FIGURE 4:**
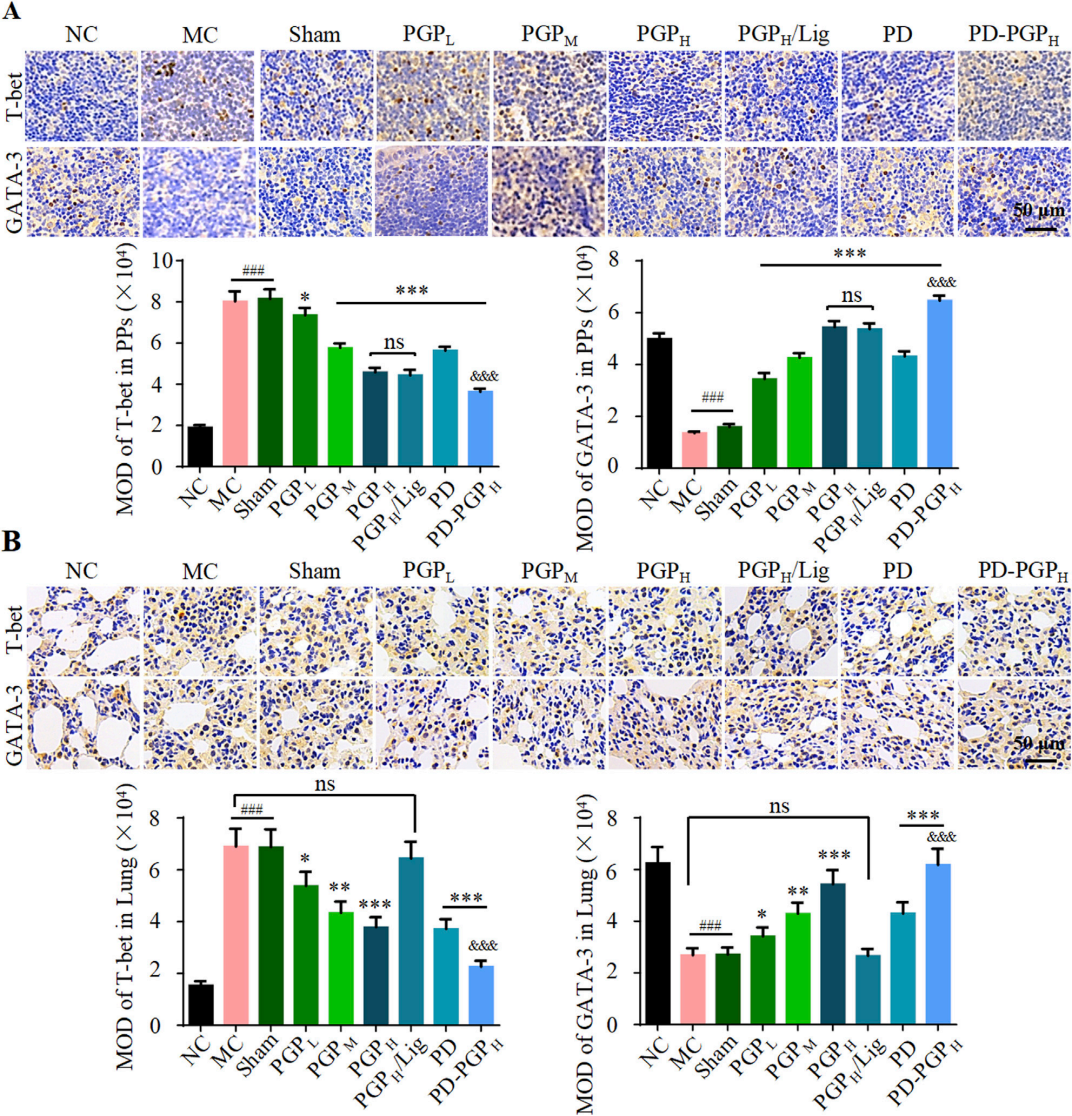
Representative images (A1 and B1) and MOD analyses (A2 and B2) of IHC staining T-bet and GATA-3 expression in PPs **(A)** and lung tissues **(B)** of CB rats, after 90 min intestinal perfusion of PGP (groups PGP_L/M/H_), PD (group PD), and PD-PGP (groups PD-PGP_H_), with (group PGP_H_/Lig) or without mesenteric lymphatic vessel ligation. T-bet and GATA-3 expression in PPs and lung tissues from normal (groups NC), model (group MC), and sham operated (group Sham) rats were set as the comparison (Original magnification ×200. Data are expressed as mean ± SD, with data obtained from 10 randomly selected fields. Data are expressed as mean ± SD (*n* = 6); ^*^ *p* < 0.05, ^**^ *p* < 0.01 and ^***^ *p* < 0.001 versus MC group; ^###^ *p* < 0.001 versus NC group; ^&&&^ *p* < 0.001 versus PD group).

The authors apologize for these errors and state that this does not change the scientific conclusions of the article in any way. The original article has been updated.

